# An Unexpected Case of *Opisthorchis felineus* Infection Revealed during Liver Transplantation

**DOI:** 10.3390/pathogens12081003

**Published:** 2023-07-31

**Authors:** Valentina D Mangano, Daniela Campani, Andrea Cacciato Insilla, Barbara Coco, Maria Angeles Gomez Morales, Maurizia Brunetto, Giuseppe La Rosa, Alessandra Ludovisi, Paolo De Simone, Fabrizio Bruschi

**Affiliations:** 1 Dipartimento di Ricerca Traslazionale e Nuove Tecnologie in Medicina e Chirurgia, Università di Pisa, 56126 Pisa, Italy; valentina.mangano@unipi.it; 2 Dipartimento di Patologia Chirurgica, Medica, Molecolare e dell’Area Critica, Università di Pisa, 56126 Pisa, Italy; 3 Unità Operativa di Anatomia Patologica 1, Azienda Ospedaliera Universitaria Pisana, 56126 Pisa, Italy; 4 Unità Operativa Epatologia, Azienda Ospedaliera Universitaria Pisana, 56126 Pisa, Italy; 5 Dipartimento di Malattie Infettive, Istituto Superiore di Sanità, 00161 Roma, Italy; 6 Unità Operativa Chirurgia Epatica e Trapianto di Fegato, Azienda Ospedaliera Universitaria Pisana, 56126 Pisa, Italy; 7 Programma Monitoraggio delle Parassitosi, Azienda Ospedaliera Universitaria Pisana, 56126 Pisa, Italy

**Keywords:** *Opistorchis*, liver transplant, parasitic infection

## Abstract

A man with hepatitis B infection was admitted to Pisa University Hospital for hepatological evaluation, which revealed multiple cystic lesions and suggested a cirrhotic evolution. Treatment with Entecavir 0.5 mg/day was started, resulting in rapid viral load suppression and alanine aminotransferase normalization. After 10 years, imaging documented a single nodule of hepatocellular carcinoma (HCC), and a robot-assisted nodule resection was performed. One year later, HCC recurrence prompted orthotopic liver transplantation, during which the patient died because of the sudden rupture of the donor’s organ and rapid multiorgan deterioration before retransplantation. During post-mortem liver examination, adult worms were evidenced within large biliary ducts, suggesting infection with *Opisthorchis* or *Clonorchis* spp. flukes. Sequencing of the *ITS2* locus, following PCR amplification of DNA extracted from liver tissue, revealed 100% identity with the reference sequence of *O. felineus*. Infection of the patient with *O. felineus* was confirmed by the presence of specific IgG detected by ELISA in the patient’s sera. Two major alkaline phosphatase serum levels peaks observed during the first two years of antiviral therapy support the hypothesis that *O. felineus* infection worsened liver function. This case report highlights the importance of a very careful screening of parasitic infections in solid organ transplantation candidates.

## 1. Introduction

Human infection with zoonotic fish-borne trematodes of the Opisthorchiidae family occurs through the consumption of raw or undercooked fresh-water fish (Cyprinoids) harboring cystic metacercariae in their muscle [1]. After ingestion the metacercariae excyst in the duodenum or jejunum and migrate to the bile duct where they mature to adult worms that remain viable for more than 10 years [2,3]. Adult hermaproditic worms reproduce and lay eggs that are released in biliary ducts and stool. When human stool contaminates fresh water, the snail intermediate host ingests eggs that release the miracidia which develop in sporocysts, rediae and finally cercariae. Cercariae are released in water, where they are able to free-swim and penetrate the fish intermediate host where they incist. Human infection is often associated with liver disease and has been strongly implicated in the pathogenesis of cholangiocarcinoma [4,5,6]. Diagnosis is based on microscopic examination of stool specimens for detection of eggs. However, the high similarity of morphological features of eggs from different species hampers species identification in most cases. Adult flukes might be recovered during surgery allowing distinction between *Opistorchis*, and *Clonorchis* spp. based on size and testes shape. Serological and molecular assays can be employed for laboratory diagnosis but are not commercially available. Treatment involves the administration of praziquantel or albendazole. Three species are mainly involved, namely *Opisthorchis viverrini*, *O. felineus*, and *Clonorchis sinensis*: *O. viverrini* is endemic in mainland South East Asia (Cambodia, Laos, Thailandia, Vietnam), *O. felineus* in Europe and Russia, and *C. sinensis* in Russia and East Asia (China, Korea, Taiwan, Vietnam) [7]. In Italy, the first two cases of human infection were reported in 2003 [8]. From then to 2021, eight outbreaks (207 cases) and some individual cases of confirmed infections have been reported. All of the infected persons had consumed raw fillets of tench (*Tinca tinca*) fished from two lakes (Bolsena and Bracciano) located in Central Italy [9,10,11,12]. In 2022, a new outbreak occurred in Central Italy, which is now under study (C. Papalini, personal communication).

## 2. Case Presentation

A man diagnosed with Hepatitis B virus (HBV) infection underwent his first hepatologic evaluation almost 40 years after diagnosis, when he accessed the Hepatology Unit of Pisa University Hospital, Italy. The main clinical and laboratory parameters are reported in Table 1. The ultrasound scan of the upper abdomen reported multiple cystic lesions (≤44 mm in diameter) and was suggestive of a cirrhotic evolution with initial signs of portal hypertension. Given the multifactorial nature (viral and dysmetabolic) and the advanced stage of liver damage (Supplementary Information), a treatment with Entecavir (ETV) 0.5 mg per day was started, resulting in a rapid viral load suppression and alanine aminotransferase normalization. Interestingly, two major but self-limiting peaks of alkaline phosphatase (ALP) levels occurred within the first two years of antiviral treatment, followed by a progressive decrease within the normal range (Supplementary Figure S1). After almost 10 years, imaging examinations carried out as part of routine follow-up documented a 45 mm single nodule of hepatocellular carcinoma (HCC) located in the subcapsular region of VIII segment considered susceptible for surgical treatment. The patient refused orthotopic liver transplantation (OLT) and underwent a robot-assisted nodule resection with complete objective response at the Computed Tomography (CT) performed one month after surgery. Six months later, alpha-fetoprotein serum levels increased up to 1071 ng/mL and a CT scan documented a local recurrence of HCC. The patient agreed to OLT, resulted eligible and underwent surgery the following month. The surgical procedure was complicated by the sudden rupture of the donor’s organ during the early phases of liver graft revascularization. The patient was maintained in anhepatic phase while the liver from another donor was successfully found but died due to rapid multiorgan deterioration before retransplantation could be attempted. 

**Figure 1:**
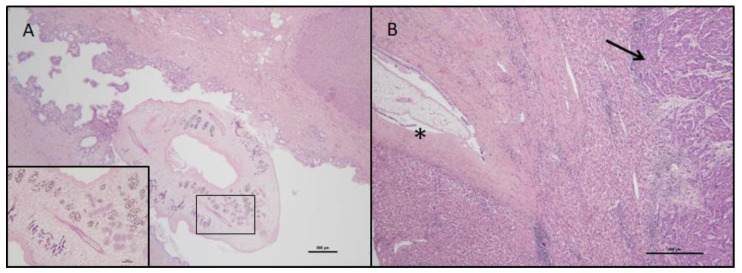
Liver histological sections showing adult flukes. The figure shows patient’s liver histological sections stained with hematoxylin-eosin. In Panel (**A**) (20× magnification, box at 100× magnification) a cross-section of an adult fluke can be observed within the common bile duct. In Panel (**B**) (40× magnification) a cross-section of an adult fluke can be observed within a secondary intrahepatic bile duct (asterisk) close to hepatocellular carcinoma (arrow).

The native liver of the patient was examined during post-mortem examination. The liver weighed 1550 g and measured 20 × 17.5 × 11.5 cm with smooth surface, a necrotic-hemorrhagic neoplasia measuring 9 × 4.5 × 3 cm was present in the V segment, serous cysts ranging from 2 cm to 6 cm were present in the V-III-II segments, and smaller bile ducts appeared focally dilated. Histological examination documented an incomplete cirrhosis, biliary hamartomas, and a poorly differentiated, grade III, angioinvasive, hepatocellular carcinoma. Adult worms were evidenced within the large bile ducts, at the hilum, and in a segmental bile duct suggesting an infection with liver flukes of the Opistorchidae family. The epithelium of the large ducts containing flukes appeared hyperplastic and partially lost (Figure 1).

**Figure 2:**
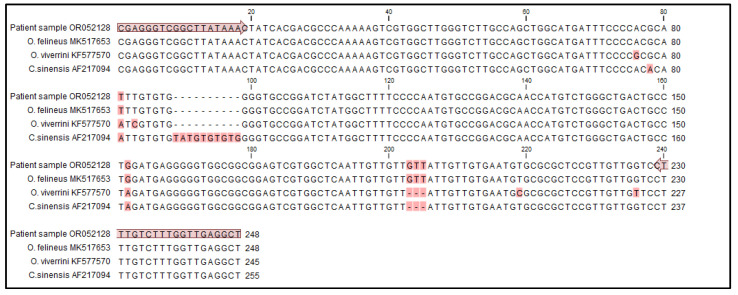
Identification of *Opistorchis felineus* by *ITS2* PCR amplification and sequencing. The alignment compares the *ITS2* partial sequence amplified by the patient sample with sequences of *O. felineus* (MK517653), *O. viverrini* (KF577570), and *C. sinensis* (AF217094) retrieved from NCBI database. The primers OP1 forward and OP2 reverse are shown using arrows and polymorphic positions are highlighted in red. The patient’s sequence shows 100% identity match with *O. felineus* (MK517653).

Molecular species identification was conducted via a PCR amplification of the Opistorchidae *ITS2* locus and sequencing of the product. DNA was extracted from two positive sections of formalin-fixed paraffin-embedded liver tissue of 10 μm thickness using Tissue and Hair Extraction Kit (Promega, Madison, WI, USA) following manufacturer instructions. PCR amplification was performed using an in-house protocol and the OP1-OP2 primer pair [13]: the reaction was carried out in a total 30 μL volume using 2× HotStarTaq Master Mix (Qiagen, Hilden, Germany), 5 pmoles of both primers (OP1 forward: 5′-CGAGGGTCGGCTTATAAAC-3; OP2 reverse: 5′-AGCCTCAACCAAAGACAAAG-3′), and 10 μL of the purified DNA sample; amplification occurred in a Veriti 96-Well Thermal Cycler (Applied Biosystem, Waltham, MA, USA) for 35 cycles (95 °C for 30″, 62 °C for 30″, 72 °C for 30″), plus a pre-step at 95 °C for 15 min and a post-step at 72 °C for 3 min. Purified DNA from *O. felineus*, *O. viverrini*, and *C. sinensis* were used as PCR positive controls. The PCR products were separated by capillary electrophoresis (Qiaxcel, Qiagen). The patient’s PCR product was purified and sequenced by Macrogen Europe B.V. The sequence was compared with that of *O. felineus*, *O. viverrini,* and *C. sinensis* collected from the NCBI database (MK517653, KF577570 and AF217094, respectively) using CLC Main Workbench Version 20.0.4 (Qiagen). The patient’s sequence showed 100% identity with the reference sequence of *O. felineus*, therefore, identifying this pathogen as the worm observed in liver biliary ducts. *ITS2* partial sequence was submitted to GenBank with accession number OR052128 (Figure 2). 

To obtain further evidence of infection by *O. felineus*, specific IgG antibodies were measured in two plasma samples collected at the time of HCC diagnosis (S1) and HCC recurrence (S2). *O. felineus*-specific IgG antibodies were measured using a previously described in-house ELISA protocol employing adult worms excretory/secretory antigens and a 1/100 dilution of the test serum [14]. The assay has 90.27% specificity and 100% sensitivity for the diagnosis of *O. felineus* infection in areas of low endemicity. Both samples resulted positive for anti-*O. felineus* IgG (S1, ELISA Index = 52.3%; S2, ELISA Index = 61.4%).

## 3. Conclusions

The present case report describes the accidental finding of adult flukes inside the large biliary ducts of a patient with HBV chronic infection who underwent OLT surgery and their identification as *O. felineus* through molecular and serological assays. Retrospectively, it is difficult to establish whether *O. felineus* infection might have been responsible for a worsening of the liver function. However, the observation that ALP serum levels presented two major peaks during the first two years of antiviral therapy may support such a hypothesis. Indeed, if most *O. felineus* infections are asymptomatic or mild, chronic infections, which are frequent in endemic areas, may lead to severe symptoms, including marked biliary tract changes, hepatomegaly pancreatitis, and cholangiocarcinoma [9,10,11,15,16]. Actually, a strong association between *O. felineus* infection and hepatobiliary pathology was recently demonstrated in an endemic area of Western Siberia [17].

To our knowledge, only one previous publication describes a similar case to that of the present report. In 2009, Melling and colleagues [18] reported a case of a patient who was diagnosed with primary biliary cirrhosis and underwent OLT in Hamburg, Germany, and whose explanted liver post-surgery pathological analysis showed histological features of opisthorchiosis. Although differentiation between *O. felineus* and *O. viverrini* was not possible, *O. felineus* was considered the most likely involved species since the patient had lived for 50 years in Kazakhstan. 

The patient herein described was not only unaware of his infection with the liver fluke, similarly to the one described by Melling and colleagues, but no risk factor for opisthorchiasis, such as frequent raw fish consumption, was revealed in the patient’s history, and routine laboratory analyses did not indicate possible parasitic infections. Therefore, the patient underwent OLT without particular precautions, such as chemoprophylaxis. On the other hand, liver donors could also be inadvertently infected, as recently reported [19]. Such reports, however rare, highlight the importance of a very careful screening of parasitic infections in solid organ transplantation candidates, especially when donors come from an endemic area as, in humans, flukes can survive for more than 10 years, and infection can remain asymptomatic [2,3].

## Figures and Tables

**Table 1:** Patient’s laboratory and clinical parameters over time.

Parameter	Time from Admission			
	Baseline	1.2 Years	9.7 Years	10.3 Years
Weight	90	97	89	89
BMI (kg/m^2^)	24.9	26.9	24.6	24.6
AST (U/L)	89	39	19	19
ALT (U/L)	67	20	8	8
GGT (U/L)	151	31	23	27
ALP (U/L)	137	166	73	86
Bilirubin (mg/dL)	0.68	0.59	0.69	0.77
INR	1.19	1.11	1.05	1.03
Albumin	3.7	4.3	4.3	4.5
PCHE	4096	6185	9740	9672
Platelets (×10^9^/L)	118	139	179	174
AFP (ng/mL)	75.4	2.4	109.9	1071
Ca 19.9 (kU/L)	281	77.1	28.8	37
Cholesterol (mg/dL)	256	205	178	185
HOMA index	6.4	2.8	0.9	1.0
HBV-DNA	2.51 × 10^6^	nd	nd	nd
HBsAg	257	89.1	nd	nd
Transient Elastography (kPa)	21.3	13.8	5.5	na

The table shows the patient clinical and laboratory parameters at admission, at 1 year of follow up, at the time of HCC diagnosis and at the time of HCC reoccurrence. Legend: AFP, alpha-fetoprotein; ALP, alkaline phosphatase; ALT, alanine aminotransferase; AST, aspartate aminotransferase; BMI: Body-mass index; GGT, gamma-glutamyl transferase; HBV, Hepatite B virus; HBsAg, HBV s antigen; HOMA, homeostasis model assessment index; INR, International normalized ratio; PCHE, Pseudocholinesterase.

## Data Availability

*ITS2* partial sequence was submitted to GenBank with accession number OR052128. Further data are available upon reasonable request to the corresponding author.

## Supplementary Materials

The following supporting information can be downloaded at: https://www.mdpi.com/article/10.3390/pathogens12081003/s1, Supplementary Information: clinical and laboratory evaluations at time of admission; Figure S1: On-treatment kinetics of patient’s main laboratory parameters.
