# Supplementary material for: An Unexpected Case of Opisthorchis felineus Infection Revealed during Liver Transplantation
Source: Pathogens. 2023 Jul 31;12(8):1003. doi: 10.3390/pathogens12081003 (PMC10459204; doi:10.3390/pathogens12081003)
Supplement: Supplementary file 1 [file pathogens-12-01003-s001.zip › pathogens-2528976-supplementary.pdf]

SUPPLEMENTAL INFORMATION

Supplementary Information: clinical and laboratory evaluations at time of admission.

The virological profile at the time of admission indicated a HBeAg negative chronic HBV infection, sustained by genotype D with presence of G1896A stop codon mutation on the Basic Core Promoter/Pre-Core region. Antibodies against Hepatitis Delta and Hepatitis C were negative, while a previous exposure to Hepatitis A virus was observed. The liver stiffness determined by transient elastography (Fibroscan, Echosens, France) was 21.3 kPa. As co-factors of liver disease, the patient reported a significant alcohol intake (up to 100 gr/die) and a history of overweight (BMI up to 29.1 Kg/m^2) that was controlled at the time of admission (BMI of 24.9 Kg/m^2). As comorbidities he had arterial hypertension that, together with dyslipidemia and insulin resistance, was suggestive of the presence of metabolic syndrome and small joints arthritis with an increased rheumatoid factor (RF=373 IU/mL) and erythrocyte sedimentation rate (ESR=68 mm/h).

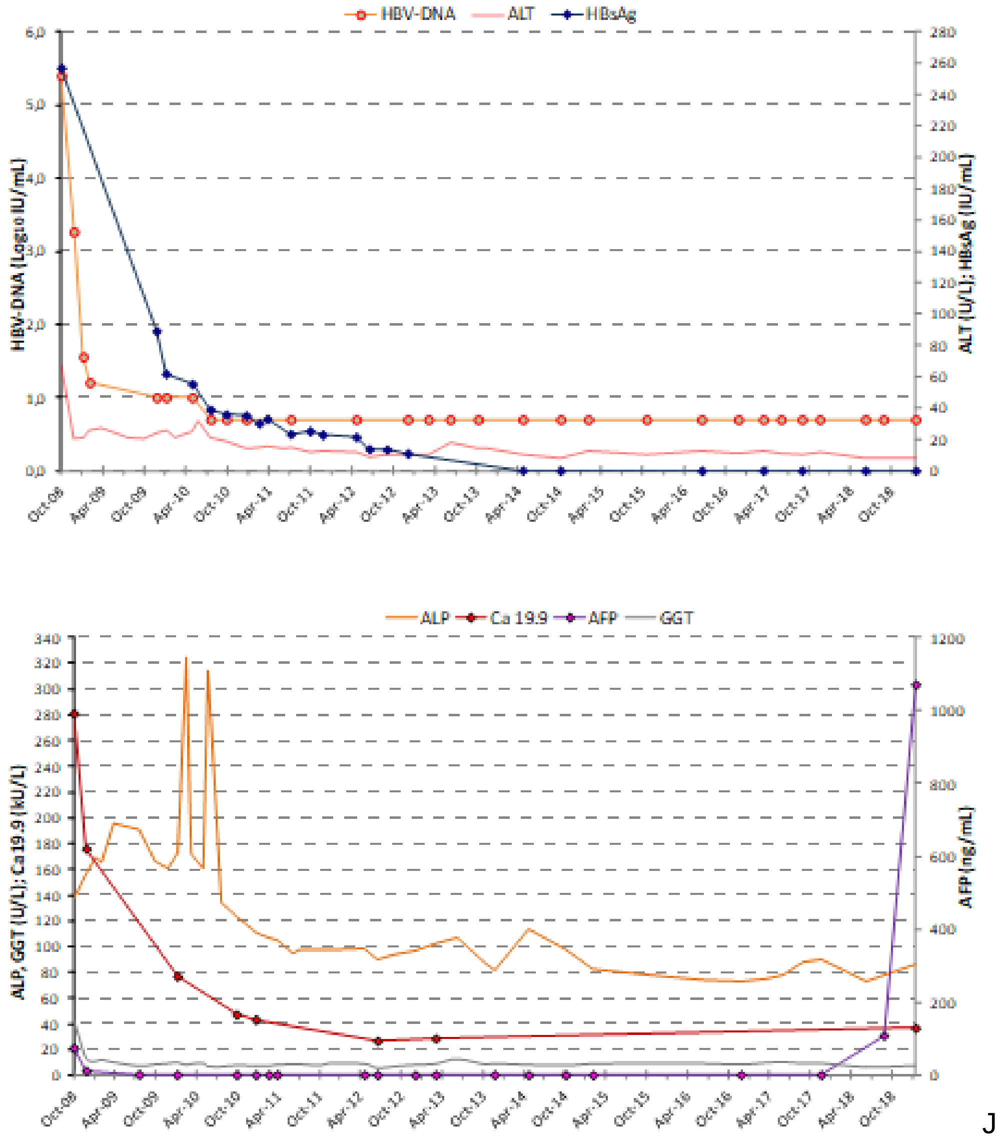

Supplementary Figure S1: On-treatment kinetics of patient's main laboratory parameters. The figure shows the patient serum levels of HBV-DNA, HBsAg (top panel) and ALP, Ca 19.9, AFP, GGT (bottom panel) during 10 years of Entecavir treatment. A rapid decline of viral load and ALT is observed. HBsAg serum levels progressively declined and its clearance was observed 6 years (2014) after the start of treatment. Persistence of mildly elevated ALP serum levels is observed during the first two-years of follow-up in spite of transaminases normalization, with two major but self-limiting peaks in March and June 2010 (325 and 314 U/L, respectively). Subsequently, ALP levels returned within the normal range and a concomitant reduction of Ca 19.9 serum levels was also observed, with a persistent normalization from 2012. AFP serum levels showed a sharp increase at HCC diagnosis and progression (2018).
